# Critical bloodstream infection caused by *Chromobacterium violaceum*: a case report in a 15-year-old male with sepsis-induced cardiogenic shock and purpura fulminans

**DOI:** 10.3389/fmed.2024.1342706

**Published:** 2024-03-26

**Authors:** Xueqing Wang, Yunliang Tu, Yingqun Chen, Huilin Yang, Minghua Luo, Yanyan Li, Lei Huang, Hua Luo

**Affiliations:** ^1^Department of Intensive Care Unit (ICU), Peking University Shenzhen Hospital, Shenzhen, Guangdong, China; ^2^Department of Microbiology Laboratory, Peking University Shenzhen Hospital, Shenzhen, Guangdong, China; ^3^Department of Pathology, Peking University Shenzhen Hospital, Shenzhen, Guangdong, China

**Keywords:** *Chromobacterium violaceum*, sepsis, purpura fulminans, cardiogenic shock, VA ECMO

## Abstract

*Chromobacterium violaceum* (*C. violaceum*) is a gram-negative bacillus that is widespread in tropical and subtropical areas. Although *C. violaceum* rarely infects humans, it can cause critical illness with a mortality rate above 50%. Here, we report the successful treatment of a 15-year-old male who presented with bloodstream infection of *C. violaceum* along with sepsis, specific skin lesions, and liver abscesses. Cardiogenic shock induced by sepsis was reversed by venoarterial extracorporeal membrane oxygenation (VA ECMO). Moreover, *C. violaceum*-related purpura fulminans, which is reported herein for the first time, was ameliorated after treatment. This case report demonstrates the virulence of *C. violaceum* with the aim of raising clinical awareness of this disease.

## Introduction

*Chromobacterium violaceum* (*C. violaceum*) is a gram-negative and facultative anaerobic bacillus that is widespread in the soil and water in tropical and subtropical regions. *C. violaceum* is an opportunistic pathogen in humans, yet precise incidence statistics are absent. Although *C. violaceum* rarely infects humans, the mortality rate for *C. violaceum* infection is above 50% (1, 2). Here, we report a case of a 15-year-old male who suffered a critical bloodstream infection caused by *C. violaceum* that progressed into sepsis-induced cardiogenic shock and liver abscesses, along with purpura fulminans (PF)-associated symmetrical peripheral gangrene (SPG).

## Case presentation

In July 2023, a 15-year-old male was admitted to the intensive care unit (ICU) of our hospital. The patient did not have a history of immunodeficiency. A week previously, he had fallen down and hurt his right limbs, and he subsequently had a fever that peaked at 41°C. His condition became exacerbated and his awareness deteriorated. Laboratory tests at the emergency room (ER) suggested severe infection accompanied by myocardial damage (Table 1).

**Table 1:** Test results of the patient infected with *C. violaceum*.

	ER (Day 1)	Day 3	Day 6	Day 12	Day 33	Day 64	Reference range
WBC (×10^9^/L)	7.71	13.21	8.82	16.82	14.71	7.21	4.1–11.0
NEU (%)	84.8	85.8	88.2	92.2	78.7	58.1	37–77
PLT (×10^9^/L)	142	26	35	103	346	646	150–407
PCT (ng/mL)	>100	>100	>100	13.8	1.41	0.13	<0.05
IL-6 (pg/mL)	>5,000	312	17.6	216	21.6	4.77	<7.0
cTnI (ng/mL)	0.307	43.9	2.62	0.39	/	/	0–0.034
ALT (U/L)	45	8,659	1,052	209	96	78	0–50
AST (U/L)	/	29,013	2,370	122	/	74	14–50
CK (U/L)	/	131,902	25,173	1,522	/	/	30–170
TB (μmol/L)	11.9	65.5	58	19.1	18.8	9.3	8.5–29.2
PTA (%)	47	18	62	71	75	85	80–120
PT (s)	19.8	41	16.7	15.5	15.0	14.1	11.0–15.0
APTT (s)	48.3	53.3	55.4	44.1	39.0	38.5	28.0–43.0
FIB (g/L)	4.2	1.67	1.61	4.55	2.51	4.14	2.00–4.00
D-dimer (mg/L)	2.23	>20	>20	5.37	0.88	0.33	0–0.50
Lac (mmol/L)	3.6	>20	2.3	1.7	1.6	/	0.5–1.7
IS	10	83	6	0	0	0	
LV diastole diameter (mm)	55	59	56	55	/	/	
Ejection fracture (%)	53	23	42	62	60	/	

WBC, white blood cell; NEU, neutrophil; PLT, platelet; PCT, procalcitonin; IL-6, interleukin-6; cTnI, cardiac troponin I; ALT, alanine transaminase; AST, aspartate transaminase; CK, creatine kinase; TB, total bilirubin; PTA, prothrombin activity; PT, prothrombin time; APTT, activated partial prothrombin time; FIB, fibrinogen; Lac, lactic acid; IS, inotropic score; LV, left ventricle.

**Figure 1:**
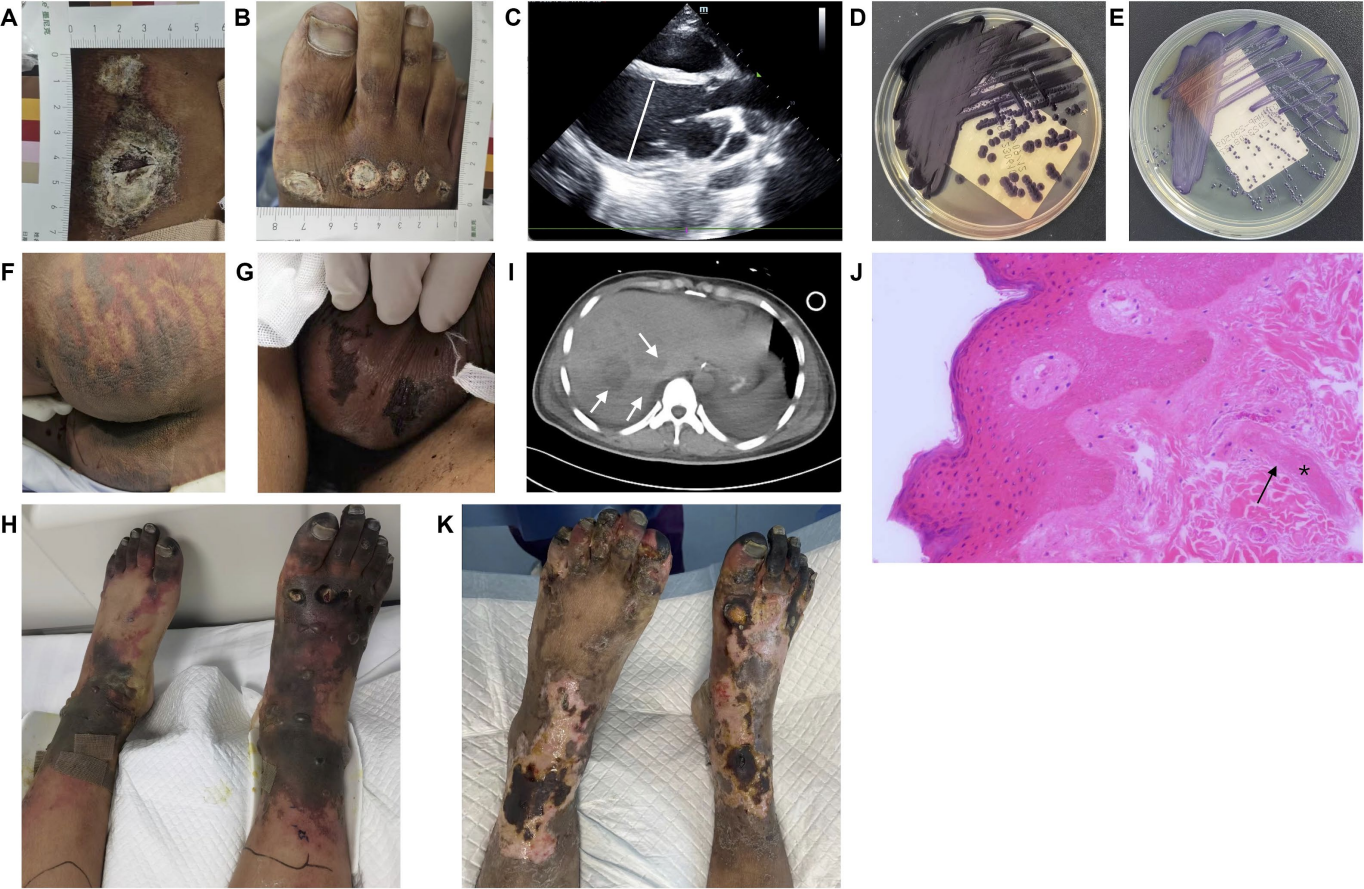
Results of physical examination, microbiological, imaging, and pathological analyses. **(A,B)** Circular skin wounds with diameters ranging from 1 cm to 5 cm, coated with white scabs. **(C)** Ultrasonic cardiogram showing an enlarged left ventricle with a diastolic diameter of 59 mm on Day 2. **(D,E)** Purple pigment secreted by *C. violaceum* cultured on MacConkey and common nutrition agar. **(F,G)** Symmetric purpura on the buttocks and scrotum. **(H)** Purpura fulminans presented with symmetric converged ecchymosis, skin necrosis, and scattered blisters, and with toes progressing to symmetrical peripheral gangrene. In addition, ulcers were present in the center of trauma wounds. **(I)** Multiple liver abscesses were detected via computed tomography (arrow). **(J)** Pathological results (100×) showed microvascular thrombosis (*) and retrogression in the squamous epithelium and vessel walls (arrow). **(K)** Partial desquamation of necrotic epidermis of the lower limbs, formation of fresh epithelium, and recession of gangrene areas limited to the toes.

When he entered the ICU ward, he was restless and had a temperature of 40°C, a heart rate of 132 beats/min, a blood pressure of 84/45 mmHg with 0.1 ug/kg/min norepinephrine, a respiration rate of 40 breaths/min, and an oxygen saturation of 100% with nasal catheter. The wounds were coated with white scabs of a regular round shape, which were mainly located beneath the right knee and on the right acrotarsium (Figures 1A,B), without significant presentation of cellulitis. Ultrasonic cardiogram (UCG) results showed a slightly enlarged left ventricle and reduced ejection fracture (EF) (Table 1). Gram-negative bacillus was detected in the blood after 13 h of culture, so meropenem (1.0 g intravenously every 6 h) and vancomycin (1.0 g intravenously every 12 h) were used as the initial empirical antibiotics.

As of 12 a.m. on Day 1, the patient had deteriorated rapidly, subsequently presenting with hypotensive shock and ventricular fibrillation. After intubation and cardiopulmonary resuscitation for 8 min, he returned to spontaneous circulation, but hypotension persisted despite treatment with the maximum vasopressor dosage. A UCG reexamination showed an enlarged left ventricle and lower EF (Figure 1C).

Venoarterial extracorporeal membrane oxygenation (VA ECMO) was conducted on Day 2. Meanwhile, *C. violaceum* resistant to penicillins, β-lactamases and carbapenems was identified in his blood sample (Figures 1D,E; Table 2). Accordingly, antibacterial treatment was adjusted to a combination of trimethoprim-sulfamethoxazole (TMP-SMX, 0.96 g orally every 6 h), amikacin (0.4 g intravenously every 12 h), and doxycycline (0.1 g orally every 12 h). With VA ECMO support, his vital signs were maintained. However, multi-organ dysfunction was revealed, including acute kidney failure, acute liver failure, and disseminated intravascular coagulation (DIC) (Table 1). Physical examination indicated newly emerged scleral icterus and progressive symmetric purpura on the buttocks, scrotum, and lower limbs (Figures 1F,G). Furthermore, *C. violaceum* was not detected in the wound secretion.

**Table 2:** Antibacterial drug sensitivity tests.

Antibacterial drug	MIC (μg/mL)	Result
Ticarcillin-clavulanate	≥128	R
Piperacillin-tazobactam	≥128	R
Ceftazidime	≥64	R
Cefoperazone-sulbactam	32	I
Cefepime	2	S
Aztreonam	16	I
Imipenem	8	I
Meropenem	≥16	R
Amikacin	4	S
Tobramycin	2	S
Ciprofloxacin	≤0.25	S
Levofloxacin	≤0.12	S
Doxycycline	2	S
Minocycline	≤1	S
Tigecycline	≤0.5	S
Colistin	≥16	R
Trimethoprim-sulfamethoxazole	≤20	S

S, sensitive; R, resistant; I, intermediate.

VA ECMO was withdrawn on Day 6 as the patient’s cardiac function improved (Table 1). After removing scabs on the wounds, ulcers were revealed in the center of the lesions. In addition, peripheral purpura worsened, especially in the lower limbs, ecchymosis converged, skin necrosis developed, blisters appeared and became confluent, and the toes progressed to symmetrical peripheral gangrene (SPG) (Figure 1H). Combined with the evidence of severe infection, shock liver, and DIC, purpura fulminans (PF) was diagnosed. The skin temperature of the feet was warm, and neither the vascular ultrasound nor computed tomography (CT) of the lower limbs demonstrated stenosis, occlusion, or thrombosis. Lab tests showed coagulation dysfunction, as well as reduced antithrombin III and protein C levels (Tables 1, 3). Enoxaparin was used to prevent thrombosis and PF-associated SPG exacerbation. In addition, the abdominal CT showed multiple liver abscesses (Figure 1I). Additionally, *C. violaceum* was identified in the sputum.

**Table 3:** Test results for antithrombin III and protein C.

	Day 3	Day 10	Day 22	Day 35	Reference range
Antithrombin III (%)	24	37	38	112	80–120
Protein C (%)	/	49	46	124	70–130

**Figure 2:**
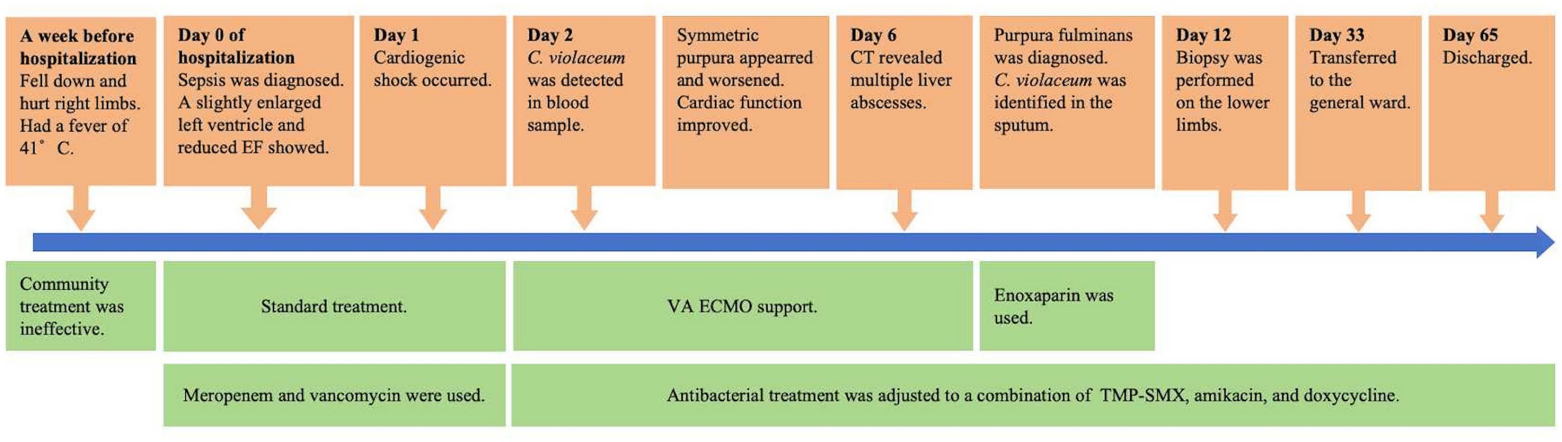
Timeline of onset, diagnosis, and treatment.

On Day 12, biopsy was performed on the lower limbs, and pathologic results indicated microvascular thrombosis with retrogression in the squamous epithelium and the vessel walls (Figure 1J). As the necrotic epidermis of the lower limbs was gradually replaced by fresh epithelium, and gangrene was limited to the toes (Figure 1K), the patient was transferred to the general ward on Day 33. He was discharged on Day 65 and admitted to a rehabilitation hospital. The brief timeline of onset, diagnosis, and treatment is shown in Figure 2.

## Discussion

Pathogenic knowledge on *C. violaceum* has increased over the decades since the first case of *C. violaceum* infection in humans was reported in 1927. However, there have still been fewer than 200 total reported cases worldwide. Despite its low infectivity, *C. violaceum* can cause critical illness with rapid progression (3). The pooled reports have revealed that *C. violaceum* infection has a mortality rate above 50%, which is sufficiently high to merit research attention (1, 2).

Given that *C. violaceum* is an opportunistic pathogen in humans, immune suppression or deficiency is a main risk factor. Approximately half of the reported cases occurred in children and immunodeficient adults. Previous studies have indicated that patients with chronic granulomatous disease or glucose-6-phosphatase deficiency are prone to *C. violaceum* infection (4–6). However, many studies have reported *C. violaceum* infection in patients with normal immunological status. Similarly, the patient in this case showed no evidence of immunological deficiency. A recent study has shown that the bacterial type III secretion system effector *Chromobacterium* outer protein C played a pivotal role in inactivating caspases to dysregulate the programmed cell death of epithelial cells, which might explain the underlying mechanism of *C. violaceum* virulence (7).

Antibacterial treatment based on a susceptibility test is the initial and most effective approach. There is a consensus that *C. violaceum* is resistant to penicillin, colistin, and most cephalosporins (1, 2, 8), but generally susceptible to fluoroquinolones, TMP-SMX, doxycycline, and amikacin (1, 2, 8). In addition, findings on *C. violaceum* resistance to carbapenems have varied (1, 8). Considering the high resistance of *C. violaceum*, in patients with severe infection, combined treatment is widely used in the acute period, and single-drug maintenance therapy for 2–3 months is recommended to prevent relapse (1).

The characteristics of *C. violaceum* infection are specific skin lesions, multi-organ abscesses, and sepsis, which were manifested in our patient. Among these characteristics, specific skin lesions include cellulitis, ulcer, purulent discharge, and necrosis. To the best of our knowledge, this is the first study to report on *C. violaceum-*induced PF. Infection-induced PF is a non-specific response caused by various organisms and has a mortality rate of 41% according to a previous study (9). Shock liver–induced acquired protein C deficiency could result in DIC, causing microvascular thrombosis of the limbs and, eventually, PF (10–12). This patient had low levels of protein C and secondary DIC, and pathologic findings of the limb lesions showed multiple thrombosis in the micro vessels, providing evidence of *C. violaceum*-induced PF. Additionally, although *C. violaceum* was not detected in the wounds, we could not eliminate the possibility that this patient’s PF was directly caused by *C. violaceum*. There is still controversy regarding the treatment. Apart from effective anti-infection treatment, other treatment options include heparin, antithrombin, zymogen protein C concentrates, or recombinant activated protein C (11). Regarding PF-associated SPG, approximately one-third of patients require amputation eventually (9). In our patient, PF-associated SPG was limited to the toes, and PF recovered as the patient was discharged. Therefore, amputation was avoided.

In this case, *C. violaceum* infection rapidly caused sepsis-induced cardiogenic shock, which has a mortality rate of >80% (13). This patient experienced progressed cardiac dysfunction and was successfully rescued with VA ECMO. In addition to our report, two previous studies have also reported the use of VA ECMO in younger children with refractory cardiogenic shock induced by *C. violaceum* (14, 15).

## Conclusion

Although *C. violaceum* rarely infects humans, its infection has a high incidence of severity, misdiagnosis, and mortality. Prior to microbiological determination, profiles of traumatic history in tropical and subtropical areas, specific skin lesions, multi-organ abscesses, and rapid progression can provide guidance for initial treatment. Clinicians should also pay attention to severe organ dysfunction and serious secondary skin presentation like PF. In critical patients, combined usage of antibacterial drugs based on susceptibility testing and proactive organ support are important.

## Data availability statement

The original contributions presented in the study are included in the article/Supplementary material, further inquiries can be directed to the corresponding author.

## Ethics statement

Written informed consent was obtained from the individual(s), and minor(s)’ legal guardian/next of kin, for the publication of this case report.

## Author contributions

XW: Writing – original draft, Formal analysis, Conceptualization. YT: Writing – review & editing, Resources, Data curation. YC: Writing – review & editing, Formal analysis. HY: Writing – review & editing, Resources. ML: Writing – review & editing, Resources. YL: Writing – review & editing, Resources. LH: Writing – review & editing, Supervision. HL: Writing – review & editing, Supervision.
