# Supplementary figures and images for: Critical bloodstream infection caused by Chromobacterium violaceum: a case report in a 15-year-old male with sepsis-induced cardiogenic shock and purpura fulminans
Source: Front Med (Lausanne). 2024 Mar 26;11:1342706. doi: 10.3389/fmed.2024.1342706 (PMC11002164; doi:10.3389/fmed.2024.1342706)

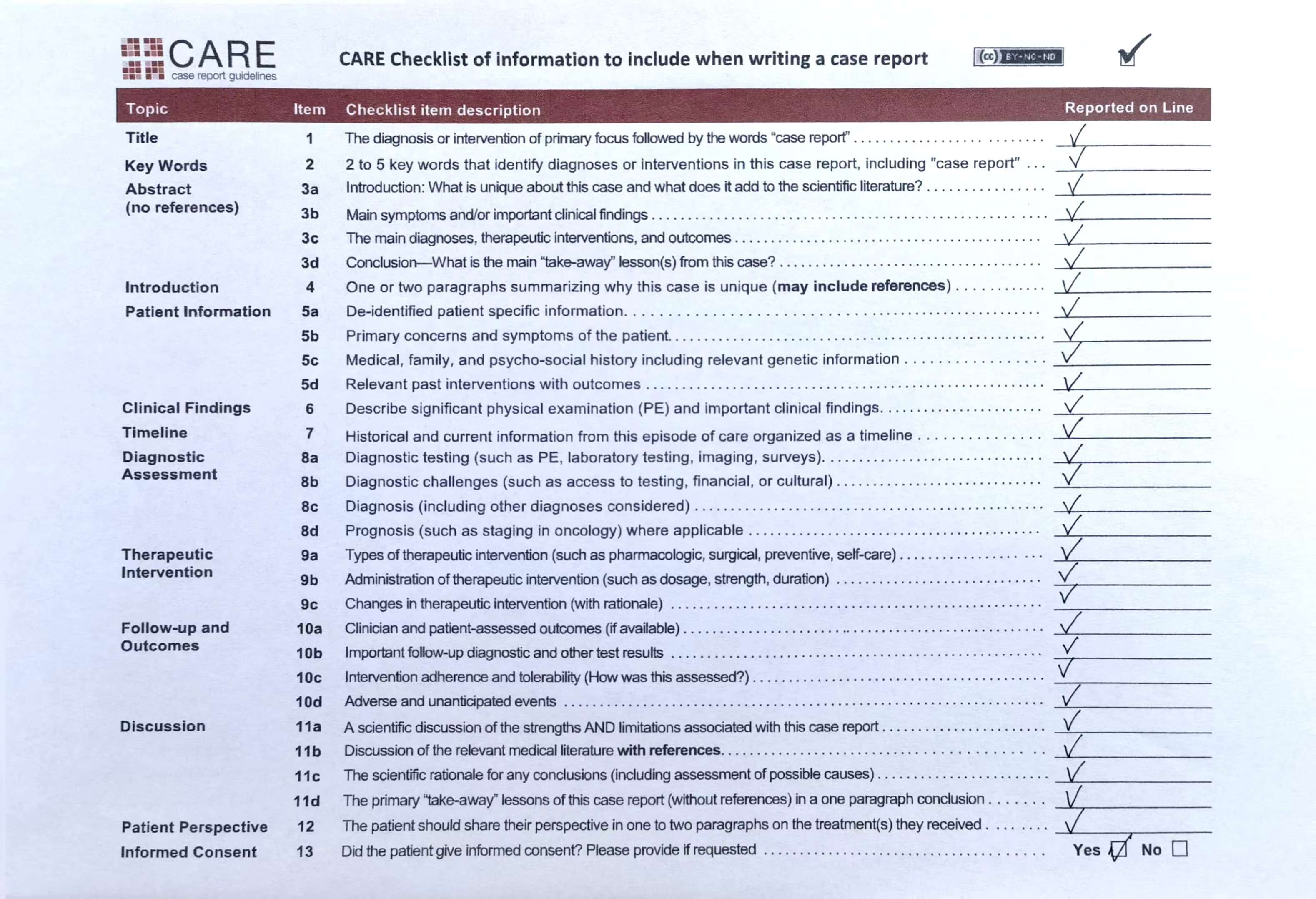

Supplement: Supplementary file 1 [file Image_1.JPEG]

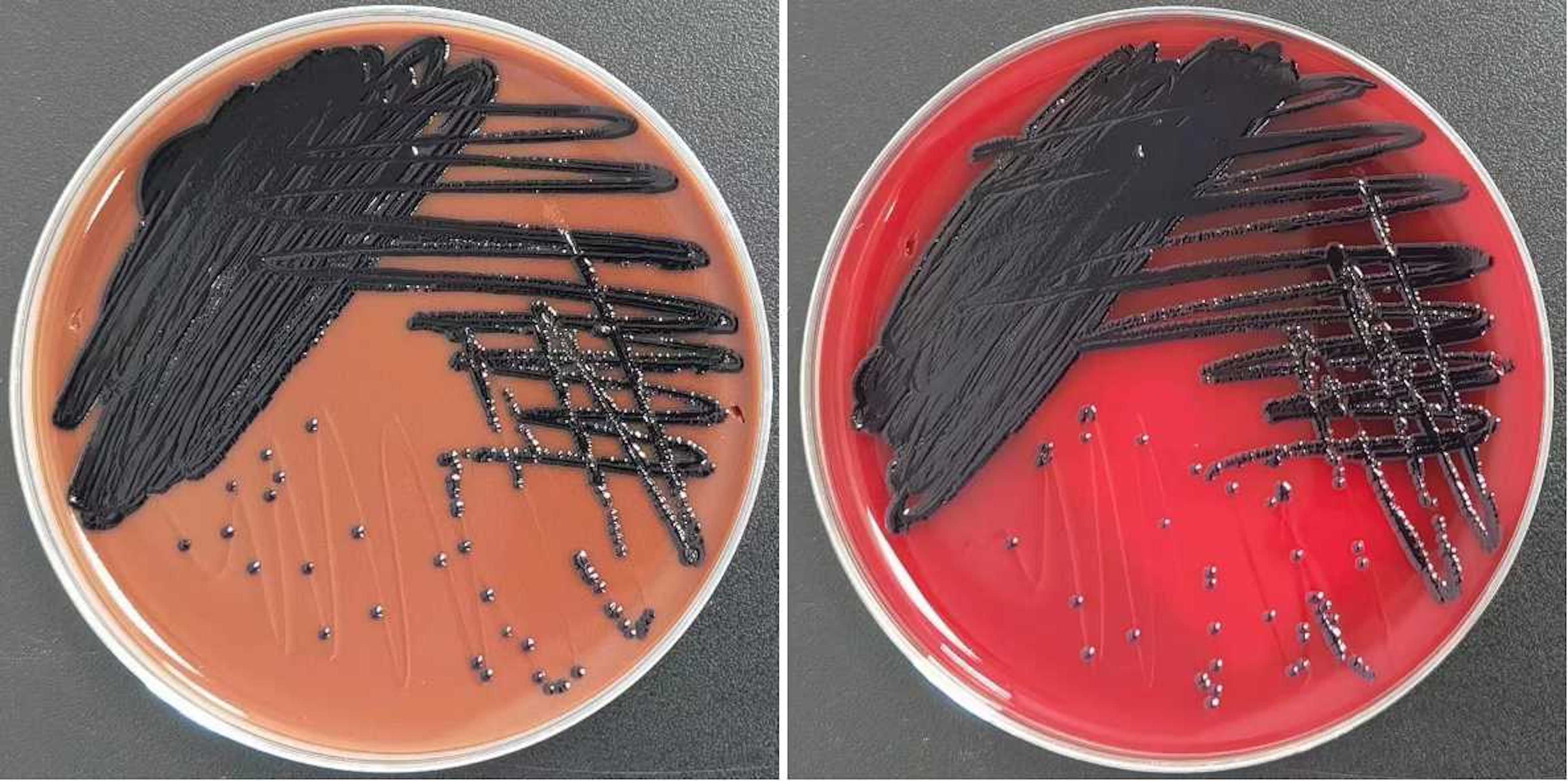

Supplement: Supplementary file 2 [file Image_2.JPEG]
